# The use of decision aids on early detection of prostate cancer: views of men and general practitioners

**DOI:** 10.1111/hex.12451

**Published:** 2016-02-18

**Authors:** Annelies Engelen, Joke Vanderhaegen, Hendrik Van Poppel, Chantal Van Audenhove

**Affiliations:** ^1^ KU Leuven LUCASKU LeuvenLeuvenBelgium; ^2^ Department of Development and RegenerationKU LeuvenLeuvenBelgium; ^3^ KU Leuven LUCASKU LeuvenLeuvenBelgium

**Keywords:** decision aid, implementation, prostate cancer, shared decision making

## Abstract

**Background and objective:**

While decision support tools such as decision aids can contribute to shared decision making, implementing these tools in daily practice is challenging. To identify and address issues around the use of decision support tools in routine care, this study explores the views of men and general practitioners on using a DA for early detection of prostate cancer.

**Methods, setting and participants:**

Group discussions and semi‐structured interviews were carried out with 43 men and 16 general practitioners familiar with a previously developed decision aid. Data were analysed using qualitative description.

**Results:**

Views on using the decision support tool could be classified into four categories: no need for decision making, need for support, perceived benefit and practical barriers. For each category, several underlying themes could be identified that reflect the absence or presence of prerequisites to successful decision support delivery.

**Discussion and conclusion:**

While men and general practitioners generally have positive attitudes to shared decision making, for both parties attitudes such as not agreeing that there is a decision to be made and doubts on the beneficence of using DAs were identified as factors that may hinder the use of a DA in clinical practice. Participants formulated strategies to support the use of DAs, mainly supplementing DAs with short tools and investing in both training programmes and large‐scale awareness raising of the general public.

## Introduction

On the topic of early detection (ED) of prostate cancer (CaP), shared decision making (SDM) is advised.^1^, ^2^, ^3^, ^4^ SDM entails patients and clinicians sharing the best available evidence when facing a decision and patients being supported to consider options as well as to achieve informed preferences.^5^ Translating this process into practice is not straightforward, especially when the evidence related to benefits and harms is complex, as is the case for ED of CaP.^6^, ^7^ To support patients and physicians in SDM, decision aids (DAs) have been developed. DAs are evidence‐based tools that prepare people for participation in making specific and deliberated choices among health‐care options by providing them with information and by supporting them in clarifying and expressing their personal wishes and values.^8^

Research shows that DAs have a beneficial impact on several aspects of decision making: increasing knowledge, value‐based decision making and SDM.^8^, ^9^, ^10^, ^11^, ^12^, ^13^, ^14^, ^15^ Realizing these advantages in daily practice requires a DA to be used outside of the research context. There are, however, few reports of successful long‐term implementation of DAs in clinical practice. Also, research on how to successfully organize the delivery of decision support is scarce.^16^, ^17^ Obstacles preventing patients and medical specialists from using DAs have been described and include general barriers such as time constraints and more context specific barriers such as the complexity of the available information.^1^, ^2^, ^11^, ^17^, ^18^, ^19^, ^20^, ^21^ While our knowledge of these factors improves, it has yet to be translated into implementation strategies that meet the needs of care providers and receivers.^18^

Our research focuses on factors influencing whether a DA on ED of CaP will be used in daily practice. This topic is characterized by high stakes and equivalent options that are difficult to balance, well suited for using a DA. We opted for an empirical, qualitative research method, taking into account the richness and variability of views brought forward by individuals and groups.^22^

## Methods

### Instrument, study design and sample

A qualitative study was conducted, consisting of group discussions with men aged 50 years and more eligible for ED of CaP and interviews with GPs in Flanders, Belgium. Methods follow the consolidated criteria for reporting qualitative research (COREQ).^23^, ^24^ Prior to data collection, participants had access to a DA on ED of CaP, ‘Making the Choice’, previously developed in line with international IPDAS‐quality criteria and in collaboration with the GPs participating in this study.^25^ This comprehensive instrument contains information in Dutch on the (dis)advantages of (not) opting for ED and provides support in clarifying and communicating preferences. It was available for participants as a booklet and as a website.^26^

To increase study participation and stimulate future DA implementation, we opted to purposefully limit our study participants to early adopters, that is potential users who are motivated to adopt an innovation and who can play an important role in stimulating adoption by other potential user groups. Therefore, we selected two participant groups: (i) GPs that showed an active interest in using DAs in clinical practice and (ii) men that were interested in or had questions about ED of CaP. We chose to include GPs active in both rural and city regions because the proximity of universities and the subsequent possibility of frequent involvement in research implies that GPs of the latter group may have a different view on novel evidence‐based evolutions such as the use of DAs than GPs of the first group.

Together with the Belgian association of GPs (Domus Medica), we organized information sessions for GPs on SDM and the use of DAs. Sessions took place throughout Flanders. At the end of each session, GPs were asked whether they were interested in testing the DA in clinical practice. The 36 GPs that answered positively were contacted for participation in this study. Eventually, 16 GPs participated in individual telephone interviews. Non‐participating GPs cited time constraints as a reason for opting out. In parallel, we contacted 50 clubs and societies to invite eligible men for participation in our study. These sociocultural clubs and societies all bring senior citizens together for various leisure activities. Five clubs and societies located in the Northern and central parts of Belgium *(Dophei Vosselaar, KWB Herent, OKRA Vosselaar, Senioren Leuven and Sint‐Sebastiaansgilde Vosselaar)* responded positively and disseminated our invitation to their participants. Eligibility criteria for participating men were as follows: (i) being 50 years or older and (ii) being interested in or having questions about ED of CaP. Men interested in participation contacted the research team directly or through the president of their club. We eventually arrived at a sample of 43 men participating in group discussions that took place in locations provided by the five involved clubs.

All participants received information on the use of DAs and on the purpose of the study. Afterwards, all men and GPs who used the DA gave their verbal and written consent to participate in the interviews or group discussions. All participants were informed that they could withdraw from the study at any time. No financial compensation was given. Prior to interviews (GPs) and group discussions (men), participants were asked to complete a questionnaire to collect demographical data as well as data concerning medical practice (GPs) or decision‐making characteristics (men) (Box 1).

Box 1 **Characteristics of participating GPs and men**

**Table:** 

Characteristics of participating GPs	Participants (*n* = 16)^a^	Characteristics of participating men	Participants (*n* = 43)
Gender		Year of birth	
Male, *n*	6	Mean	1951
Female, *n*	9	Range	1938–1964
Year of birth		Partner relation	
Mean (range)	1964	Partner relation, *n*	42
Range	1946–1977	No partner relation, *n*	1
Number of years practicing		Education	
Mean	23.3	High school graduate or less, *n*	23
Range	5–38	College graduate, *n*	20
Patients per week in consultation		Employment status	
Mean	98	Employed, *n*	29
Range	55–150	Unemployed/retired, *n*	14
Hours in consultation per week		Using the Internet b	
Mean	35.5	A few times a month, *n*	1
Range	8–60	About once a week, *n*	3
How often does a patient show an interest in ED of CaP?		More often than once a week, *n*	36
Less often than once a year, *n*	1	Did you already receive information on ED of CaP?	
More often than once a year, *n*	0	No, *n*	17
More often than once a month, *n*	12	Yes, *n*	26
More often than once a week, *n*	2	Preference in decision‐making style	
		Patient (me) alone, *n*	1
		Patient after considering GP opinion, *n*	20
		Patient and GP, *n*	14
		GP after considering patient opinion, *n*	4
		GP alone, *n*	1

a One GP who participated in the interviews (*n* = 16) did not fill out the questionnaire.

b Three men did not answer this question.

### Data collection and analysis

All five group discussions (men) and 16 individual telephone interviews (GPs) were conducted in October 2013. Group discussions involved 5–13 participants and lasted about 120 min. Individual interviews with GPs lasted about 30 min. Using a semi‐structured discussion guide, the interviews and group discussions progressed from broad, open‐ended questions to narrower questions with specific probes to clarify issues if needed.^22^ All interviews and group discussions were conducted by AE, a junior biomedical researcher with experience in conducting qualitative research. AE was supported in this process by JV, a senior researcher who assisted in one of the group discussions. Data collection and analysis were supervised by CVA, a professor with substantial experience in conducting qualitative research. The interviewer had not been in contact with the study participants prior to the start of this study. She participated in the development of the DA used in this study and has witnessed the difficulties of implementing these tools in daily practice.

**Figure 1:**
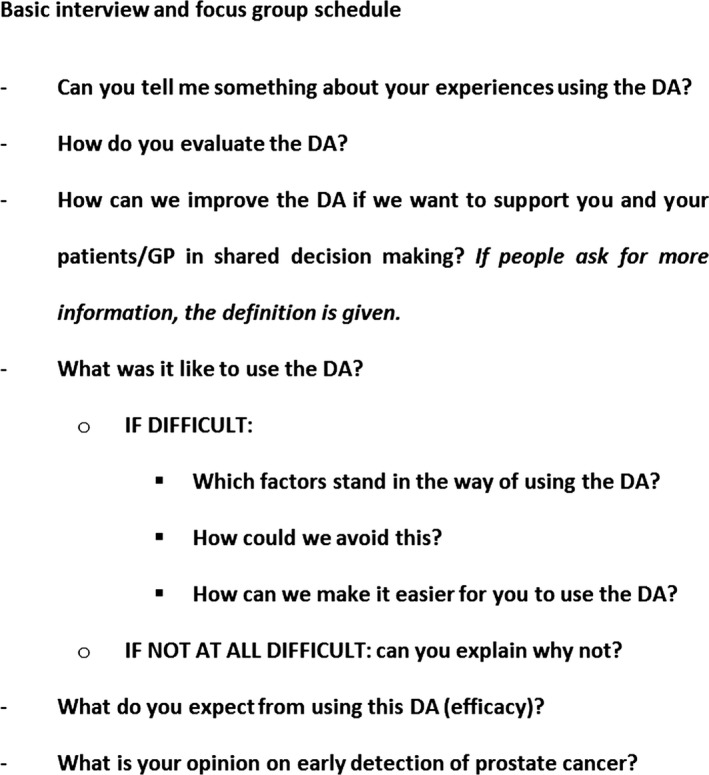
Basic interview and focus group schedule.

Interviews and group discussions focused on three major topics: (i) the evaluation of the instrument and its use in consultation, (ii) factors that may hinder or facilitate implementation and the realization of positive effects and (iii) views on the ED decision (Figure 1). Views of GPs and men relating to the evaluation of the developed tool as such will be presented elsewhere. The topic guide was developed by the research team and was based on experiences in previous studies on the development and evaluation of DAs.^9^, ^27^ The questions were intended to stimulate conversation on the perspectives of men and GPs on using a DA on ED of CaP. Participants were encouraged to talk freely about their experiences and views. It was explained that the purpose of the interview or group discussion was not to reach agreement and that there were no ‘bad’ answers or comments. No repeat interviews were carried out. The focus groups and interviews were recorded, transcribed verbatim and managed using NVivo10 software (QSR international Pty Ltd., Doncaster Australia). Field notes made during the interviews and group discussions were used to inform data analysis. At several points during each interview or group discussion, the interviewer presented a brief summary of the main ideas and asked participants whether they would like to make changes or additions. Data were reported anonymously to maintain confidentiality.

Qualitative description was used to analyse and report the data collected.^28^ In a first step of thematic analysis, the most important topics and concepts were defined by open coding of each group discussion and interview separately, soon after it had taken place. Each information unit of the transcripts was examined for emergent themes in relation to the issues explored and labelled accordingly. We identified *in vivo* codes and explored ideas for the advancement of more abstract codes. As further data were analysed, each transcript was revisited and the coding was revised. The labels assigned to the ideas emerging from the transcripts were brought together, inductively categorized and refined through an iterative process. After thematic analysis by AE, the accounts of GPs and men were compared to assess what we could learn from their different perspectives. During the whole process of analysis, whenever it was unclear how transcripts should be coded, this was resolved through team discussions. The sample size was sufficient to reach theoretical saturation as the final group discussions and interviews did not change the study themes.

## Results

We identified four categories related to using the DA: (i) no need for decision making, (ii) need for support, (iii) perceived benefit and (iv) practical barriers. All categories contain several underlying themes. All categories were identified for GPs and men alike. All categories and themes are described below alongside relevant data extracts.

### No need for decision making

While in each group discussion at least one man mentioned that ED of CaP has both benefits and drawbacks, men generally reported no need for decision support because they felt there was no decision to be made. Men highlighted the benefits of ED and saw no reason not to test. They also admitted being worried about CaP or about staying healthy in general. Several men said that their partners advised them to get tested. GPs, on the other hand, generally reported that there is no decision to be made. They did not favour ED of CaP because, to them, it has too many drawbacks and too few advantages.

You just have to look at it step by step and start with the beginning – not being worried yet and thinking ‘what if?’ . … The next time I have a blood test, I'll have the PSA value determined and then we'll see what the next steps are and how we can deal with it. At that point you can still make a decision on what to do with this information. Thinking about these things now is way too soon. [Group discussion men 2]

The aspects of overtreatment and making men worried… I am reluctant. You do not have to leave everything up to fate, but it has to be useful. You should not create too much unrest in people. The collateral damage should not be too high and in this case, I think it is. [Interview GP 1]

The different views of men and GPs led to different expectations or goals concerning ED of CaP. Specifically, the protesting attitude of men led them to expect their GPs to test them proactively. GPs reported being aware of these expectations, but also mentioned that it would be better not to bring up ED of CaP during the consultation to avoid inadvertently giving men the idea to get tested. Some GPs discussed how they felt thwarted in their efforts to keep silent about this topic by the urologists and partners who advice men to get tested.

On the level of the PSA test and the rectal exam, which have no negative consequences really, … I think the decision is not difficult. I go to the doctor every year … to get a checkup and each year I get a rectal exam and sometimes a PSA test. I think it is normal that [the GP] does this, just like it is for cholesterol and blood pressure. [Group discussion men 1]

What's often the case is that their family or friends tell them that they should get tested for CaP. Urologists sometimes say that as well. Then you're left without backing, as a GP. [Interview GP 9]

When men discussed how their GPs did not spontaneously bring up ED of CaP, the overall sentiment was one of indignation. Some men and GPs described how men would ask their GP to test them for CaP when he did not do so spontaneously. Confronted with these expectations and questions, many GPs admitted testing men despite their misgivings, either habitually and spontaneously or only in response to a direct question.

First man: ‘For every blood test, there is a form on which the GP checks the boxes for [all the tests that need to be done]. Well, check the box for this test. And that's it. Second man (indignant): I have had to ask my GP, because otherwise he didn't check the box! … . I think it should be [checked]. [ … ] First man: Maybe that is something you researchers need to work towards, with GPs: “If you have a patient that is older than 50, you should always order a PSA test when you do a blood test.”’ [Group discussion men 2]

There are patients who really want to know. … I do think that my patients expect it of me … . that I meet their expectations . … Men are willing to listen to my reservations about testing, but in the end they want it [to be tested]. [Interview GP 14]

While men and GPs did express how they prefer SDM in general, men were not eager to participate in decision making on ED of CaP – a feeling expressed by men and acknowledged by GPs. Men furthermore mentioned in most group discussions that they do not consider this decision to be important and that they do not like to talk about CaP. While some GPs did mention how they inform men on the topic of ED of CaP and involve them in decision making, it became apparent that GPs and men currently generally do not engage in SDM on ED of CaP

First man: It touches on the manliness of men. … . Second man: With these possible side effects of impotence and incontinence … . Third man: it is a delicate business. First man: First you have a man and the consequence could be that he is no longer a man, and that's why there is a taboo. [Group discussion men 3]

The PSA test, for most men, is like ‘I want it’, or ‘I don't want it’. I do not have the impression that there are many men who first want to read about it, and think about it and deliberate about it. Probably there are other topics for which it is true that patients want to think and read about it. [Interview GP 13]

### Need for support

Whereas the participating GPs and men generally reported that they experience no need for decision making, their accounts did show that both parties experience a need for support. Men generally expressed a need for support in communicating about ED of CaP with their GPs. Both men and GPs perceived ED of CaP as a difficult topic on which men lack understanding. Both parties expressed a need for patient‐centred information. In addition, GPs expressed a need for support in the process of informing men on ED of CaP. In line with their generally positive attitude towards testing, in all group discussions men pointed out that it would be great if there would be a tool that encourages testing. GPs, on the other hand, mentioned that there is a need for neutral DAs that reach men before strong positive perceptions of testing are formed and a decision is made.

It is good that [the DA] will be there – because otherwise you're 100% dependent on what your GP will tell you. Also, on the internet you'll only find contradictory information that doesn't help you very much. So I think it's very good [that it exists]. [Group discussion men 5]

Of course, [ED of CaP] is a difficult topic. You have positive and negative test results and then you conduct another test and it can again be positive or negative and then you can either treat or not treat. There is this duality in everything associated with it. [Interview GP 6]

### Perceived benefit

GPs and men indicated that DAs could have several effects and evaluated these effects differently. Members of both parties mentioned that the DA could lead to either more or less testing. A decrease in testing due to DA use was generally perceived as harmful by men and as advantageous by GPs, while a potential increase in testing was generally perceived as advantageous by men, but as harmful by GPs.

I was wondering, when I was reading it: Won't there be people who say: ‘all those disadvantages!’ and then withdraw … It's going to have downsides, people are going to say: ‘For now, everything is all right; I'm not going to rouse the sleeping giant!’ … . I'm afraid that some people might say: ‘What am I getting into? I might be worse off than when I started!’ [Group discussion men 4]

Well, I believe that if a man would be informed beforehand [on the disadvantages of testing], that this could have a good, positive influence in that it would lead to less testing. [Interview GP 5]

In addition, on the topic of SDM, some men and GPs were convinced that using the DA could foster communication, while others disagreed. GPs mentioned that using the DA could reduce time investment for SDM. On the topic of information, men and GPs reported that the DA could help users to understand the complex nature of ED of CaP. Furthermore, men described the tool as a good source of information and GPs stated that the tool would support them in their role as information giver. While some men mentioned that using a DA could make it easier to arrive at a decision, other men and GPs felt that using the DA would make it harder to make a decision.

### Practical barriers

GPs discussed they often experience time pressure in daily practice. Men acknowledged this and mentioned that time constraints might hinder DA use. Both parties described how using short tools aimed at fostering SDM in a time‐efficient manner alongside or instead of more comprehensive DAs, during or outside of the consultation, could address this barrier to DA use.

To what extent do GPs still have the time? Because I think they experience a lack of time. To have a conversation on the topic with every patient over the age of 50 – I don't think they can just say: ‘I am going to talk about it with every 50‐year‐old’. [Group discussion men 2]

There could be a summary or fiche … on our desks. If a man comes with a question, we can go over these points. … If somebody really wants to know more, then you can refer to the website and then you can tell him to have a look at it in his own time. If somebody says that he really doesn't want all the information and just asks you to do the test … well, you have provided some information. [Interview GP 8]

Furthermore, many men and GPs pointed out that they have no experience in using DAs and that this might hinder DA use. GPs advised to organize training sessions to support them in using DAs. In addition, some GPs and men doubted whether men would be capable of using DAs. Both parties pointed out that it may be challenging for patients to search for an online DA and to use it efficiently. To address this potential barrier to DA use, men advised in all group discussions to ask GPs to deliver the DA to interested men. Yet, some GPs pointed out that this might be difficult to achieve given the time pressure. A solution to this problem mentioned by men and GPs alike was to provide a short folder or poster in the waiting room. However, several men pointed out that this does not guarantee that all men find the DA since not all men regularly visit their GP. Alternatively, men and GPs described how it would be helpful if men's awareness on the existence of DAs and where to find them could be increased by large‐scale awareness‐raising campaigns.

Giving GPs the opportunity to practice with [the DA] so that they can browse it quickly. … For example during LOK meetings [i.e. periodically organized meetings for physicians as part of permanent education], … Practical training, concrete: A patient sits in front of you, this is his question, he asks for a blood test, for a PSA test, how are you going to deal with this? [Interview GP 4]

I think you should [increase awareness] by using the media. There are these shows on the television like X [informative program] where they raise issues such as [using DAs] – maybe they can focus on this topic, for once. So that it is brought out in the open [Men GD 4]

## Discussion

In this study, we explored the views of men and GPs on using a DA on ED of CaP, allowing us to identify factors influencing its use in clinical practice. Four categories of adoption factors were identified: (i) no need for decision making, (ii) need for support, (iii) perceived benefit and (iv) practical applicability.

On the topic of ED of CaP, it became clear that many GPs and men experienced no need for decision making. Moreover, the opposing attitudes of men and GPs to ED of CaP lead to a clash of expectations. Men expected to get tested and reacted indignantly when their GP did not do so spontaneously while GPs remarked that it is difficult for them to deal with men's unquestioningly positive attitude towards testing. In the process of SDM, it may occur that both GPs and men bring their opinions and values into the consultation. When preferences of GPs and men differ, as in this case, this may negatively affect their relationship, both parties' feelings and the likelihood of achieving true SDM – certainly when men are unlikely to openly disagree with their GPs.^29^

Yet, in general, GPs and men did express a need for support in communication, understanding information and information giving. In fact, both parties admitted that men are currently strongly under‐ or misinformed on ED of CaP. Because ED in truth is a complex topic with advantages and disadvantages that need to be weighed in decision making, men's unquestioningly positive attitude towards testing is indicative of mis‐ or underinformation.^1^, ^30^ As ED has long been portrayed in an unquestioningly positive way in Belgian general media, this misinformation may in part be explained by previous exposure to protesting advertising. With regard to DA implementation, misinformation may hinder DA uptake by reinforcing the opinion that there is no decision to be made. Without care providers and users agreeing that a balancing act is in order when considering a specific medical topic, neither party will see any merit in doing a balancing act together. Indeed, views on the potential benefit of using the DA were mixed. To tackle misconceptions and foster informed decision making, using DAs is a valid approach. Yet, the results of this study make it clear that it is difficult to achieve effective use of DAs when both parties initially feel there is no decision to be made.^8^

The accounts of GPs and men showed that whether tests are conducted is often strongly influenced by the patients' desire to get tested. Since many men have an uninformed positive attitude towards testing, this may result in tests being ordered for men who would not have opted for ED if they would have been more correctly and fully informed on the topic.^31^ In addition, the DA may reach men when they have already decided in favour of testing, thus limiting its potential impact on the decision or decision‐making process. As such, the lack of information explains why many men experienced no need for decision making – a barrier to DA use – and why there is a need for support to put the prevailing misconceptions right. As a consequence, it becomes important to research means to provide information to men in a way that is not influenced by misinformation as a barrier to information giving. Essentially, this would imply providing information in a way that is less dependent on men's desire to access the information, such as providing information on a large scale in general media.

On a practical level and in accordance with prior research, our results indicate that the practical applicability of DAs is severely curtailed by time constraints^20^ To address this barrier, men and GPs repeatedly proposed to opt for short tools that can be used in a time‐efficient manner during the consultation and can either replace or complement comprehensive DAs. Several research groups have already experimented with short decision support tools.^32^, ^33^, ^34^ An example is the recent development of Option Grids: short one‐page tools that can be used during consultation to optimize the SDM process.^32^ Prior research about clinical topics such as breast cancer or head and neck cancer has shown that using Option Grids can contribute to SDM and can support GPs in delivering information.^35^, ^36^ Research also shows that interventions targeting patients and health‐care professionals are more promising than those targeting one or the other.^14^ Additionally, in Belgium, DAs are still novelties, unfamiliar to most health professionals and patients. This lack of experience can be addressed by information and training sessions aimed at GPs on using DAs efficiently.^19^ Research shows that multifaceted interventions that include both efforts to educate health‐care professionals and the use of DAs are promising in promoting the adoption of SDM in clinical practice.^15^ On the patients' side, our study highlights the importance of increasing patients' awareness on the existence of DAs and where to find them.

This study has some limitations. Firstly, it focuses on the use of a DA in one specific complex medical context by participants who were interested in using a DA or in ED of CaP and who had the cognitive abilities to understand and discuss the decision aspects. The specific themes identified in this study may not be present as such in other medical contexts or in different user groups. Yet, we do believe that the broad categories of no need for decision making, need for support, mixed feelings on potential benefit and practical applicability also affect the implementation of DAs in other decisional contexts. Secondly, the recruitment strategy employed does not allow for providing information on the number of eligible men that chose not to participate to the study and why.

Thirdly, since it proved unfeasible to organize group discussions because of time constraints, we conducted telephone interviews with GPs. Both methods may lead to a different depth and width of insight in the participants' views and experiences, which may have influenced our results. Also, interviewing about a care innovation is susceptible to social desirability bias. However, participants were reminded explicitly that they could freely speak their mind. An important strength of this study is that both men and GPs had access to a DA that was specifically designed for them, which allowed for focused and informed communication on one topic and resulted in a broad overview of factors influencing the effective use of decision support tools. Future research should focus on the extent to which the lack of need for decision making, perceived benefit and practical barriers influence DA implementation. Also, research should be done on a DA development strategy that takes the pre‐existing preferences and attitudes of the target audience into account. We believe that the DA development process should be preceded by an assessment of pre‐existing attitudes and potential practical barriers to DA use.

## Conclusion

The use of DAs on ED of CaP is influenced by multiple adoption factors. A lack of need for decision making and passive role preferences hinder patient participation in decision making. Yet, both GPs and men indicate a need for informational and communicative support. At the same time, the perceived time investment associated with using a DA and a lack of experience hinder the use of DAs. To overcome barriers to the use of a DA, we follow the recommendations of men and GP and call for an increased focus on the development and practical evaluation of short decision support tools that can be used in a time‐efficient manner during the consultation. Yet, to achieve successful DA implementation we recommend that changes be made not only on the level of the tools used, but also on an attitudinal level. This calls for health professionals and patients to be supported in using tools and to be informed on aspects of the subject matter of the tool and on SDM. Training programmes for health professionals that are provided in an accessible, time‐efficient way should be an integral part of any decision support strategy. Additionally, large‐scale awareness raising can set patients' misconceptions right and can increase patients' awareness on the existence of DAs and where to find them.

## Funding source

This project was funded by The Vlaamse Liga tegen Kanker (Flemish League against Cancer). The funding source had no role in the design and conduct of the study or in the collection and analysis of the data, nor in the drafting of the manuscript for publication.

## Conflict of interest

The authors declare no conflict of interest.
